# The Home of Recovery

**Published:** 1909-08-07

**Authors:** 


### THE HOME OF RECOVERY.

The following important announcement appeared in the newspapers on August 2 :—

"Mr. Ernest Frederick Schiff, of Carlos Place and Warnford Court, has presented, through Mr. Mayo Robson, to the managing committee of the Home of Recovery, of which H.R.H. Princess Louise is president and the Earl of Lytton is chairman, the magnificent sum of £100,000. Part of this sum has been expended upon the purchase of a charming and suitable property within seventeen miles of London, to which the patients will be conveyed by motor ambulance straight from their beds in the hospitals. The remainder will be added to the funds already in the hands of the Committee and will serve as a permanent endowment of the home. This announcement is appropriately made public to-day on the anniversary of the death of Mr. Alfred George Schiff—the brother of the generous donor—in whose memory the gift has been made. The Institution will be called 'The Schiff Home of Recovery' to perpetuate the name of one who was widely known during his life for his sterling qualities and for many philanthropic acts performed in a silent and unostentatious manner."

The Home of Recovery for Surgical Patients was founded in 1907 under the presidency of H.R.H. Princess Louise, Duchess of Argyll. The Messrs. Schiff have for many years been much respected members of the Stock Exchange, and the late Mr. A. G. Schiff, who left an estate valued at over £570,000, was a generous and unostentatious friend to a large number of medical and other charities. "The Schiff Home of Recovery" will enable surgical patients when they leave the hospitals in which they have undergone operations to continue the treatment which will equip them for a satisfactory resumption of their customary occupations. Hospital surgical patients are frequently discharged on account of the pressing claims of others who are waiting for admission before they have sufficiently recovered. The Schiff Home will accelerate surgical treatment in hospitals and permit a freer interchange of in-patients, since those who pass thither from surgical wards will be supplied with a continuance of whatever treatment may be necessary in order to bring about their complete recovery. Mr. E. F. Schiff has set a noble example to those who possess wealth. The Home of Recovery should prove a boon to the surgical in-patients of hospitals and to the hospital authorities as well, whose difficulties have long been accentuated by the absence of any large institution for recovering surgical patients.

